# Symptoms and medical resource utilization of patients with bronchiectasis after SARS-CoV-2 infection

**DOI:** 10.3389/fmed.2023.1276763

**Published:** 2024-01-08

**Authors:** Jun Wang, Jiaqi Ren, Xin Li, Juan Wang, Chun Chang, Lina Sun, Yongchang Sun

**Affiliations:** Department of Respiratory and Critical Care Medicine, Peking University Third Hospital. Research Center for Chronic Airway Diseases, Peking University Health Science Center, Beijing, China

**Keywords:** bronchiectasis, SARS-CoV-2, medical resource, symptoms, infection

## Abstract

**Background:**

The impact of COVID-19 caused by severe acute respiratory syndrome coronavirus 2 (SARS-CoV-2) on patients with bronchiectasis in terms of symptoms, self-management and medical resource utilization was unknown.

**Objective:**

To describe the impact of infection by SARS-CoV-2 on fluctuation of symptoms, self-management and medical resource utilization of patients with bronchiectasis during the pandemic of COVID-19.

**Methods:**

This was a single-center cross-sectional questionnaire study performed in Peking University Third Hospital. An online questionnaire investigation addressing the impact of SARS-CoV-2 infection on respiratory symptoms, self-management and medical resource utilization was conducted among patients with bronchiectasis during the COVID-19 surge in December 2022 in Beijing, China.

**Results:**

Five hundred patients with bronchiectasis, with 285 (57%) females, and a mean (±SD) age of 57.9 ± 15.1 years, completed the telephone questionnaire. The reported prevalence of COVID-19 was 81.2% (406/500). Of the 406 COVID-19 patients, 89.2% experienced fever lasting mostly for no more than 3 days, 70.6 and 61.8% reported exacerbated cough and sputum production respectively, and 17.7% reported worsened dyspnea. Notable 37.4% of the patients with COVID-19 experienced symptoms consistent with the definition of an acute exacerbation of bronchiectasis. However, 76.6% (311/406) of the infected patients did not seek medical care but managed at home. Of the patients who visited hospitals, 26.3% (25/95) needed hospitalization and 2.1% (2/95) needed ICU admission. Multi-factors logistic regression analysis showed that younger age (*p* = 0.012) and not using a bronchodilator agent(*p* = 0.022) were independently associated with SARS-CoV-2 infection, while a history of exacerbation of bronchiectasis in the past year (*p* = 0.006) and daily use of expectorants (*p* = 0.002) were associated with emergency visit and/or hospitalization for patients with bronchiectasis after SARS-CoV-2 infection.

**Conclusion:**

During the COVID-19 surge, the infection rate of SARS-CoV-2 in patients with bronchiectasis was high, and most of the patients experienced new-onset or exacerbated respiratory symptoms, but only a minority needed medical visits. Our survey results further underscore the importance of patients’ disease awareness and self-management skills during a pandemic like COVID-19.

## 1 Introduction

Bronchiectasis is defined as abnormal dilation of the bronchi, typically presenting with symptoms such as chronic cough with sputum production, dyspnea, and recurrent respiratory exacerbations. It represents the third most frequent chronic inflammatory diseases of the airways, after asthma and chronic obstructive pulmonary disease (COPD), and is an increasingly common disease in China, with an estimated prevalence of 174.45 (137.02, 211.88) per 100,000, which increased 2.31-fold from 2013 to 2017 (1), posing a high social and economic burden (2, 3).

Acute exacerbations (AE) of bronchiectasis are associated with increased airway and systemic inflammation (4), worse quality of life (5), progressive lung damage (6, 7) and more medical resource utilization. Respiratory viruses can be identified during exacerbations in up to 50% of patients with bronchiectasis (8, 9) and have been postulated to disturb the balance between chronic bacterial colonization and host-defense response, leading to outgrowth of bacteria and heightened inflammatory responses which resulted in acute exacerbation. The coronavirus (CoV) was one of the most common viruses detected in nasopharyngeal swab or sputum in patients with bronchiectasis experiencing an exacerbation (10).

Coronavirus disease 2019 (COVID- 19), caused by the novel severe acute respiratory syndrome CoV 2 (SARS-CoV-2), has spread rapidly worldwide since December 2019 (11). During the pandemic, the impact of COVID-19 on the management of chronic diseases has received much attention, which, for airway diseases, was concentrated mostly on risks of SARS-CoV-2 infection in patients with asthma and COPD (4, 5, 7, 12, 13), but the impact on patients with bronchiectasis in terms of respiratory symptoms, self-management and medical resource utilization is not known. A UK COVID -19 population study (13) showed that the diagnosis of bronchiectasis was associated with a risk of hospitalization (HR 1.34) and of death (HR 1.12) with COVID-19. In contrast, a nationwide retrospective cohort study in China showed that, after adjustment for age, sex, and other systemic comorbidities, patients with bronchiectasis were not more likely to need invasive ventilation, admission to intensive care unit, or to die at day 30 after hospitalization, compared with those without (6). However, because most people with COVID-19 had not been admitted to hospital, selecting only hospitalized patients for cohort entry often led to enrollment bias. Up till now, SARS-CoV-2 infection and its natural course in the population with clinically diagnosed bronchiectasis have been rarely studied.

In the early December of 2022, the strict measures for preventing COVID-19 were lifted in Beijing, and a large population experienced SARS-CoV-2 infection. Therefore, we undertook a survey to investigate the prevalence of SARS-CoV-2 infection and the symptoms, self-management and medical resource utilization in patients with bronchiectasis during this pandemic surge.

## 2 Method

### 2.1 Study design

This was a cross-sectional questionnaire study performed in Peking University Third Hospital. All subjects had been confirmed to have bronchiectasis by chest HRCT in Peking University Third Hospital. An online questionnaire investigation addressing the impact of SARS-CoV-2 infection on patients with bronchiectasis and self-management and medical resource utilization was conducted.

The study was approved by the Ethics Committee of the Peking University Third Hospital (registry M2021-428). All the procedures were performed in accordance with the guidelines of the authors’ institutional ethics committee and adhered to the tenets of the Declaration of Helsinki.

### 2.2 Criteria for inclusion and exclusion

The criteria for inclusion: patients with bronchiectasis who had visited Peking University Third hospital between 1 January 2018 and 30 November 2022; adult status (18 years or more); residence in Beijing.

The criteria for exclusion: refusal to participate in the study.

**Figure 1:**
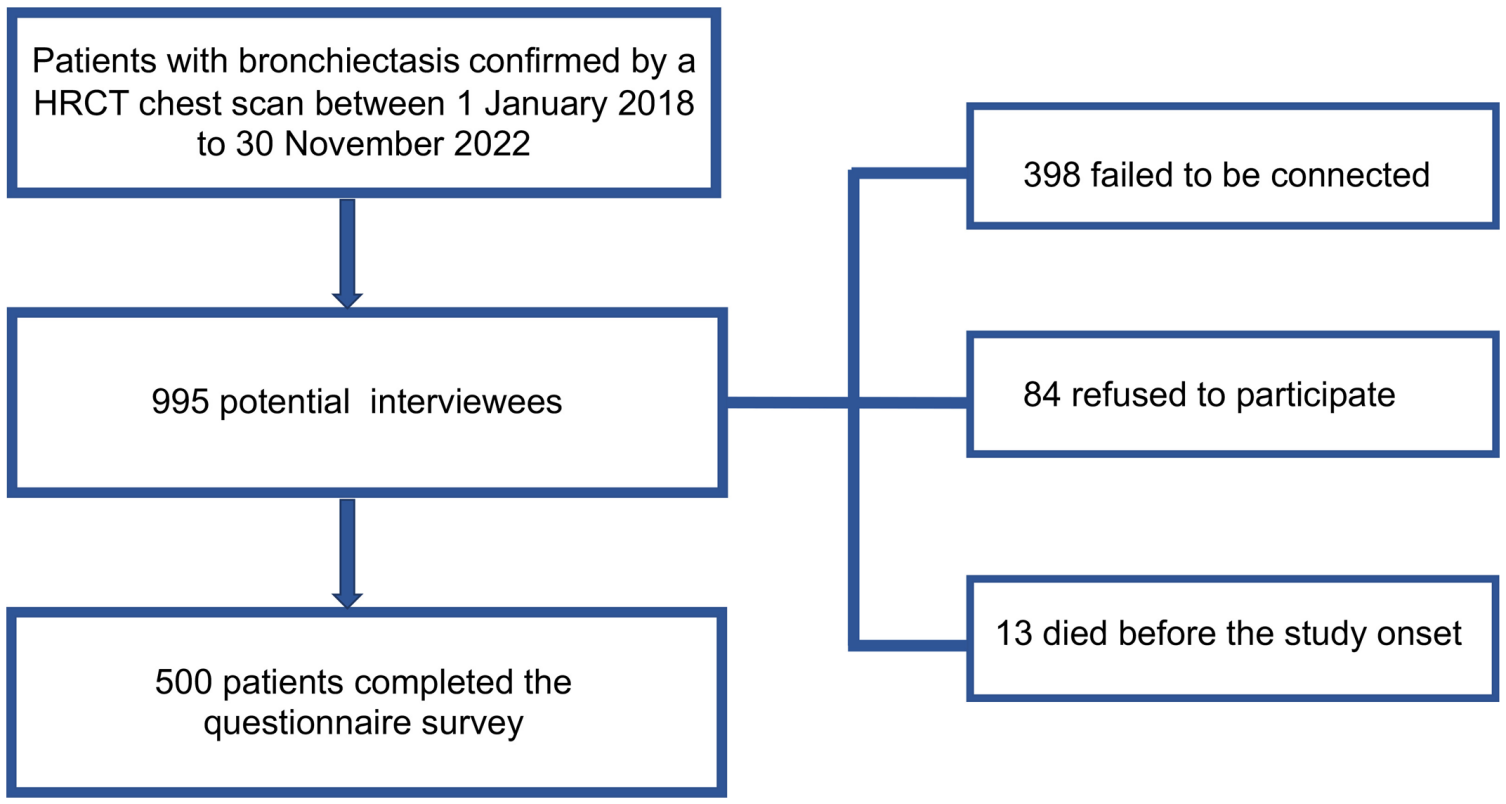
Flowchart of study. HRCT, high-resolution computerized tomography.

The survey was conducted by telephone call. Initially, 995 patients were identified as potential interviewees, of whom 398 failed to be connected, 84 refused to participate, and 13 died before the study onset. Finally, 500 patients finished the questionnaire. The flowchart of our study was shown in Figure 1. According to the principles of sampling for a cross-sectional survey, the sample size needed to be 5–10 times the questionnaire items (14). The number of questionnaire items in this study was 25, and therefore 500 participants met the needs of statistical analysis.

### 2.3 Structured questionnaire and measurements

An online administered questionnaire consisting of several parts was constructed. The introduction of the questionnaire described the background and purpose of the survey and stated that the questionnaire would be answered anonymously and voluntarily following informed consent. Basic demographic information included age, gender, body mass index (BMI), and smoking habits. Baseline data related to bronchiectasis consisted of the disease course, main manifestations, exacerbation times in the past year, underlying etiology and stable stage therapy of bronchiectasis, comorbidity and vaccination history. Questions about COVID-19 included SARS-CoV-2 infection status, methods of diagnosis, symptoms, self-management and medical resource utilization.

The symptoms of COVID-19 were defined as those emerging or aggravating on pre-existing symptoms such as fever, cough, expectoration, dyspnea (shortness of breath, chest tightness, and wheezing), loss of appetite, and fatigue.

### 2.4 Analyzed variables

#### 2.4.1 Baseline variables

The following baseline variables were analyzed: age, gender, BMI, smoking history, age at diagnosis of bronchiectasis, chronic symptoms of bronchiectasis, and pharmacological treatment of bronchiectasis.

#### 2.4.2 Exacerbation history

An exacerbation of bronchiectasis (15–17) was defined as the presence of three or more of the following symptoms worsening for more than 48 h: cough, volume and/or consistency of sputum, purulence of sputum, dyspnea and/or intolerance of exercise, asthenia and/or general malaise, and hemoptysis, as well as a need for a change in treatment, for example as the need of antibiotics, and exclusion of other causes of clinical deterioration.

#### 2.4.3 SARS-CoV-2 infection

The methods of diagnosis of SARS-CoV-2 infection included laboratory confirmation of SARS-CoV-2 by a nucleic acid test, or a positive self-administered antigen test, or consistent symptoms and epidemiology. The following variables were analyzed: the prevalence of infection of SARS-CoV-2, the symptoms (and duration) caused by COVID-19, medical visits, medicines used, hospitalization and intensive care admission.

### 2.5 Statistical analysis

Data were expressed as mean ± standard deviation or median (interquartile range, IQR) for continuous variables depending on whether or not they followed a normal distribution, while categorical variables were expressed as counts and percentages. Both parametrical (Student’s t-test for repeated measurements) and non-parametrical (Wilcoxon) tests were used to compare the quantitative variables depending on the variable distribution. In the case of qualitative variables, proportions were compared by means of the chi-square test, as well as Fisher’s exact test, where necessary. Logistic regression was used to analyze the associated risk factors. A two-tailed value of *p* of <0.05 was considered statistically significant. Missing values were not imputed. All analyses were performed using SPSS version 20 Armonk, NY.

## 3 Results

### 3.1 Baseline characteristics of the patients

Of the 500 patients who completed the telephone questionnaire, 285 (57%) were female, and the mean (±SD) age was 57.9 ± 15.1 years (Table 1). Most respondents (388/500, 77.6%) had been vaccinated against COVID-19 (Table 1). The prevalence of cough, sputum production, dyspnea and/or exercise intolerance, hemoptysis at baseline (i.e., stable stage before having COVID-19) was 62, 59.2, 12.0, and 18.0%, respectively. 140 (28%) patients reported at least one AE, of whom 32.9% (46/140) with at least one AE needing hospitalization, in the past year (Table 1). 4.6% (23/500) of the patients were treated with ICS, 14% (70/500) received long-acting *β*-agonists (LABA) or/and long-acting muscarinic antagonists (LAMA), while 4.2% (21/500) received ICS plus a LABA (Table 1). In terms of the potential etiologies for bronchiectasis, post-infection accounted for 22.8% (114/500), post-tuberculosis for 16.0% (80/500), and those with unknown causes for 61.2% (306/500).

**Table 1:** Demographic and baseline characteristics of patients with bronchiectasis.

	*n* = 500
Age (mean ± SD)	57.9 ± 15.1
Sex (male, %)	215 (43.0)
BMI (mean ± SD)	21.7 ± 6.8
Cigarette Smoking (No., %)	129 (25.8)
COVID-19 vaccination doses (mean ± SD)	3.2 ± 1.3
Chronic symptoms before lifting of COVID-19 control measures (No., %)	
No symptoms	126 (25.2)
Cough	310 (62.0)
Sputum	296 (59.2)
Hemoptysis	90 (18.0)
Dyspnea	60 (12.0)
Wheezing	57 (11.4)
Maintenance therapy (No., %)	153 (30.6)
Bronchodilators	70 (14)
ICS	23 (4.6)
Expectorants	71 (14.2)
Acute exacerbation in the past year (No., %)	140 (28)
Comorbidity (No., %)	
Hypertension	87 (17.4)
COPD	41 (8.2)
Asthma	41 (8.2)
Diabetes	43 (8.6)
Malignancy	17 (3.4)

COVID-19, coronavirus disease 2019; SD, standard deviation; BMI, body mass index; ICS, inhaled corticosteroids; COPD, chronic obstructive pulmonary disease.

### 3.2 The prevalence of SARS-CoV-2 infection

81.2% (406/500) of the patients reported infection by SARS-CoV-2, of whom 82 (20.2%) were confirmed by nucleic acid tests, 281 (69.2%) by antigen tests, and 107 (26.3%) were verified by typical symptoms and a history of close contact with family members with SARS-CoV-2 infection.

### 3.3 Symptoms and clinical course of patients with bronchiectasis after SARS-CoV-2 infection

**Figure 2:**
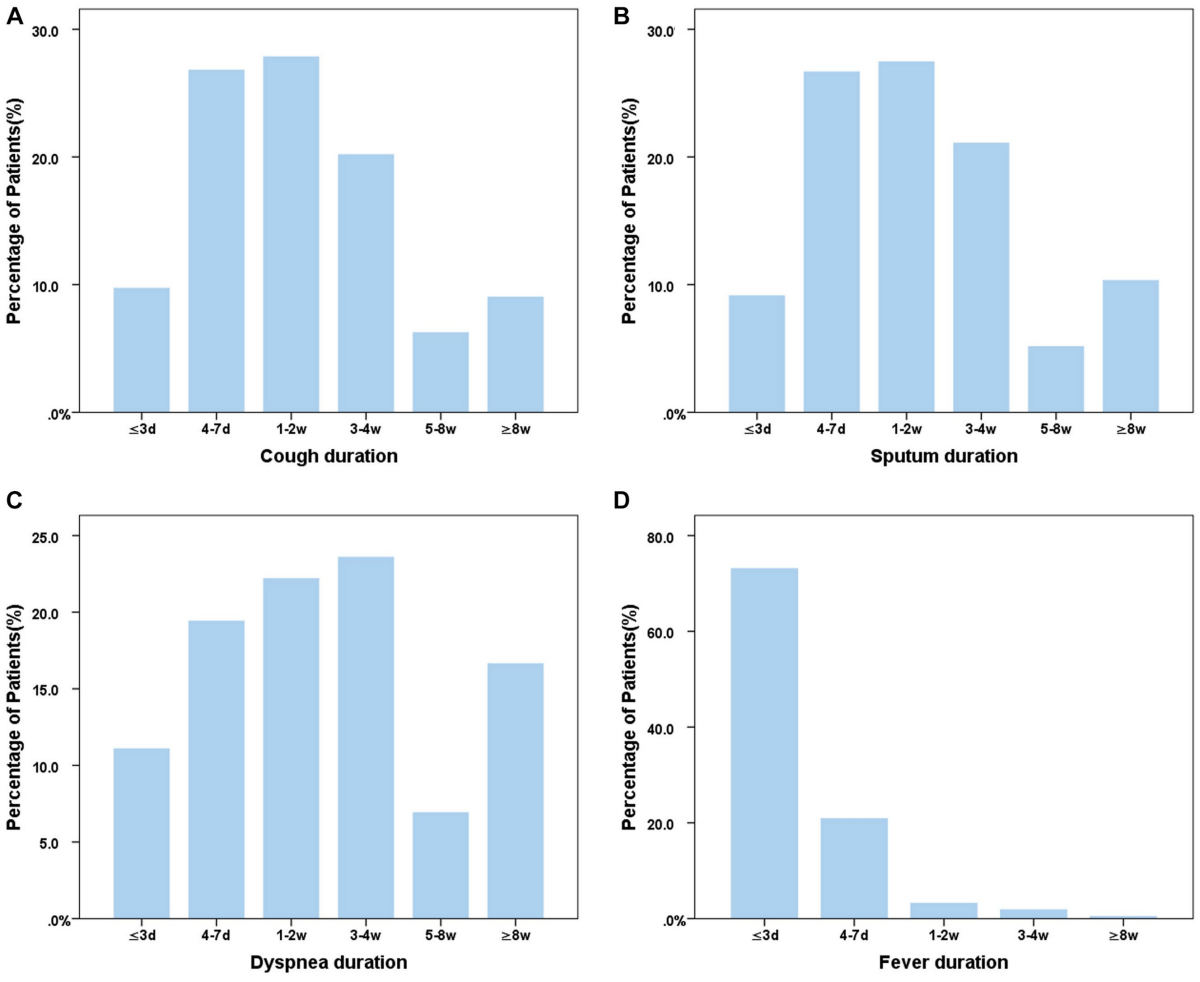
Distribution of main symptom duration in bronchiectasis patients with COVID-19. **(A)** Cough duration of bronchiectasis patients with COVID-19; **(B)** Sputum duration of bronchiectasis patients with COVID-19; **(C)** Dyspnea duration of bronchiectasis patients with COVID-19; **(D)** Fever duration of bronchiectasis patients with COVID-19. ≤3d, 4-7d, 1-2w, 3-4w, 5-8w, ≥8w means the course of symptom ≤3d, 4-7d, 1-2w, 3-4w, 5-8w, ≥8w means the course of symptoms was less than 3 days, 4-7 days, 1–2 weeks, 3-4 weeks, 5–8 weeks and more than 8 weeks, respectively.

Of the 406 COVID-19 patients, 1.47% (6/406) had no symptoms, while 70.6% experienced cough (Figure 2A), 61.8% had expectoration (Figure 2B), 17.7% complained of dyspnea (Figure 2C), and 89.2% (400/406) had fever which lasted mostly for no more than 3 days (Figure 2D). It was notable that 37.4% (152/406) of the patients with COVID-19 experienced symptoms consistent with the definition of an acute exacerbation of bronchiectasis.

### 3.4 Self-management and medical resource utilization of patients with bronchiectasis after SARS-CoV-2 infection

Of the 406 bronchiectasis patients with COVID-19, 76.6% (311/406) did not seek medical treatment but managed by themselves. Antipyretic drugs (49.1%, 153/311) and oral antibiotics (15.8%, 49/311) were the two most commonly used drugs at home. The main reason (92.6%, 288/311) for not seeking medical care was that the patients believed that the symptoms were mild and could resolve spontaneously, while the remaining (7.4%, 23/311) responded that they had difficulty in seeking medical treatment. Of the patients who sought medical care, 26.3% (25/95) needed hospitalization and 2.1% (2/95) needed ICU admission.

### 3.5 Differences in demography and clinical characteristics between COVID-19 and non-COVID-19 patients with bronchiectasis

Compared with COVID-19 patients with bronchiectasis, non-COVID-19 patients with bronchiectasis were older (62.7 Vs. 52.8 years, *p* = 0.001), with a higher prevalence of hypertension (24.5% Vs. 15.8%, *p* = 0.045), with a higher proportion of long-term drug treatment for bronchiectasis (39.4% Vs. 28.6%, p = 0.04) and bronchodilator treatment (26.6% Vs.11.1%, *p* < 0.001), Table 2. Multivariable logistics analysis including age, hypertension, long-term drug treatment and bronchodilator treatment showed that older age [0.473 (0.264, 0.846), *p* = 0.012] and using a bronchodilator agent [0.514 (0.290,0.910), *p* = 0.022] were independently negatively correlated with SARS-CoV-2 infection, Table 3.

**Table 2:** Comparison of characteristics between bronchiectasis patients with and without COVID-19.

	COVID-19 *n* = 406	Non- COVID-19 *n* = 94	*p* value
Age (mean ± SD)	56.8 ± 15.0	62.7 ± 14.8	0.001*
Sex (male, %)	175 (43.1)	40 (42.6)	0.923
BMI (mean ± SD)	21. 7 ± 7.0	21.8 ± 5.6	0.913
Cigarette Smoking (No., %)	106 (26.1)	23 (24.5)	0.676
Maintenance therapy (No., %)			
Bronchodilators	45 (11.1)	25 (26.6)	<0.001*
ICS	17 (4.2)	6 (6.4)	0.36
Expectorants	57 (14)	14 (14.9)	0.831
Acute exacerbation in the past year (No., %)	115 (28.3)	25 (26.6)	0.736
Comorbidity (No., %)			
Hypertension	64 (15.8)	23 (24.5)	0.045*
COPD	33 (8.1)	8 (8.5)	0.903
Asthma	34 (8.4)	7 (7.4)	0.768
Diabetes	36 (8.9)	7 (7.4)	0.658
Malignancy	14 (3.4)	3 (3.2)	0.901

COVID-19, coronavirus disease 2019; SD, standard deviation; BMI, body mass index; ICS, inhaled corticosteroids; COPD, chronic obstructive pulmonary disease.

**Table 3:** Risk factors for COVID-19 in patients with bronchiectasis.

Single-factor logistic			Multi-factorl logistic	
	OR with 95% CI	*p* value	OR with 95% CI	*p* value
Age(>56)	0.359 (0.209,0.616)	<0.001*	0.473 (0.264,0.846)	0.012*
Hypertension	0.578 (0.336,0.992)	0.047*		
Maintenance bronchodilators	0.379 (0.219,0.654)	<0.001*	0.514 (0.290,0.910)	0.022*

OR, odd ratio; CI, confidence interval; *p < 0.05.

### 3.6 Risk factors for emergency visiting and/or hospitalization

The demographic and clinical characteristics of COVID-19 patients and the risk factors for emergency visiting and/or hospitalization were shown in Tables 4, 5. In group comparison and univariate risk analysis, age, COVID-19 vaccination times, daily symptom of sputum, dyspnea, wheezing, and regular use of bronchodilators, expectorants, and acute exacerbations in the past year, comorbidity of COPD and diabetes were risk factors for emergency visit and/or hospitalization after SARS-CoV-2 infection. However, multivariable analysis showed that only acute exacerbation in the past year (*p* = 0.006) and long-term use of expectorants (*p* = 0.002) remained to be significant risk factors.

**Table 4:** Comparison of characteristics between patients who needed and those who did not need emergencycare and/or hospital admission.

	Emergency care and/or hospital admission (*n* = 39)	No emergency care and/or hospital admission (*n* = 367)	*p* value
Age (mean ± SD)	65.7 ± 16.0	55.8 ± 14.6	0.001*
Sex (male, %)	18 (46.2)	157 (42.8)	0.686
BMI (mean ± SD)	21. 7 ± 4.8	21.8 ± 7.2	0.991
Cigarette smoking (No., %)	14 (35.9)	92 (25.1)	0.143
COVID-19 vaccination doses (mean ± SD)	2.5 ± 1.4	3.3 ± 1.2	<0.001*
Chronic symptoms before lifting of COVID-19 control measures (No., %)			
No symptoms	4 (10.3)	100 (27.2)	0.034*
Cough	29 (74.4)	219 (59.7)	0.074
Sputum	30 (76.9)	204 (55.6)	0.01*
Hemoptysis	10 (25.6)	67 (18.3)	0.263
Dyspnea	10 (25.6)	38 (10.4)	0.005*
Wheezing	10 (25.6)	36 (9.8)	0.003*
Maintenance therapy (No., %)			
Bronchodilators	12 (30.8)	37 (10.1)	<0.001*
ICS	3 (7.7)	14 (3.8)	0.466
Expectorants	19 (48.7)	38 (10.4)	<0.001*
Acute exacerbation in the past year (No., %)	21 (53.8)	94 (25.6)	<0.001*
Comorbidity (No., %)			
Hypertension	7 (17.9)	57 (15.5)	0.694
COPD	10 (25.6)	23 (6.3)	<0.001*
Asthma	2 (5.1)	32 (8.7)	0.441
Diabetes	8 (20.5)	28 (7.6)	0.007*
Malignancy	3 (7.7)	11 (3.0)	0.127

COVID- 19, coronavirus disease 2019; SD, standard deviation; BMI, body mass index; ICS, inhaled corticosteroids; COPD, chronic obstructive pulmonary disease.

**Table 5:** Risk factors for emergency care and/or hospital admission.

	Single-factor logistic		Multi-factor logistic	
	OR with 95% CI	*p* value	OR with 95% CI	*p* value
Age (>56)	2.548 (1.176, 5.520)	0.018*		
COVID-19 vaccination doses (>2)	0.279 (0.140, 0.554)	<0.001*		
No symptoms	0.305 (0.106, 0.880)	0.028*		
Sputum	2.663 (1.230, 5.769)	0.013*		
Dyspnea	2.985 (1.350, 6.600)	0.007*		
Wheezing	3.170 (1.429, 7.034)	0.005*		
Bronchodilators	3.964 (1.853, 8.478)	<0.001*		
Expectorants	8.225 (4.035, 16.764)	<0.001*	3.818 (1.652, 8.824)	0.002*
Acute exacerbation in the past year	3.388 (1.731, 6.633)	<0.001*	2.904 (1.358, 6.212)	0.006*
COPD	5.157 (2.241, 11.870)	<0.001*		
Diabetes	3.124 (1.312, 7.439)	0.01*		

COVID- 19: coronavirus disease 2019; COPD: chronic obstructive pulmonary disease OR: odd ratio; CI: confidence interval; *p<0.05.

## 4 Discussion

There have been several studies, mostly retrospective, investigating the impact of COVID-19 on bronchiectasis in hospitalized patients (4, 6), or comparing the difference between COVID-19 patients with and without bronchiectasis (13, 18–20). However, there was a lack of study on the epidemiological and clinical data, self-management and medical resource utilization of bronchiectasis patients infected with SARS-CoV-2 during the COVID-19 pandemic. The COVID-19 pandemic resulted in the public recognition of social distancing and mitigation measures that reduced person-to person interactions. There was a significant reduction in the frequency of reported exacerbations of bronchiectasis during the lockdown period (21–23). For example, an observational, multicenter study in Spain showed that the proportion of patients without any exacerbations increased from 22.6% in the pre-pandemic period to 63.1% in the pandemic (*p* < 0.001) (22). However, after the lift of COVID-19 lockdown, the prevalence and the impact of SARS-CoV-2 infection on patients with bronchiectasis was not clear.

The present study, to our knowledge, was the first to describe the infection rate, respiratory exacerbation and medical resource utilization in patients with bronchiectasis during a COVID-19 surge in China. We found that the infection rate of SARS-CoV-2 in bronchiectasis patients was 81.6%. Of the bronchiectasis patients infected by SARS-CoV-2, 37.4% experienced symptoms consistent with the definition of an acute exacerbation of bronchiectasis. The common symptoms of bronchiectasis patients with COVID-19 included fever and new-onset or exacerbated respiratory symptoms, such as cough, expectoration and dyspnea. The duration of fever was short (≤ 3 days), while respiratory symptoms (such as cough, expectoration, and dyspnea) lasted much longer (4 days to 4 weeks). Notably, 76.6% patients did not need immediate medical care but successfully managed at home. Of the patients who sought medical care, 26.3% needed hospitalization and only 2.1% needed ICU admission. We also noted that, compared with the uninfected patients, those infected by SARS-CoV-2 were younger and were less likely to receive bronchodilator therapy.

Bronchiectasis patients with SARS-CoV-2 infection reported a wide range of symptoms on presentation. Similar to other population studies (11, 16, 24–26), fever was the most frequent symptom in our cohort. The frequency of fever (89.2%) in the present study was similar to most previous studies (11, 25, 26), but higher than the data from a system review on clinical characteristics for COVID-19 (37.0%) (16).

Cough was another common symptom in COVID-19 patients (11, 16, 25–27). The incidence of cough (71.6%) in our study was similar to that reported in other studies (11, 26, 27), but higher than the data from a systematic review of COVID-19 (25.4%) (16). The frequency of dyspnea (17.7%) in our patients with SARS-CoV-2 infection was mostly similar to, or higher than that reported elsewhere (11, 26, 27), although lower than that from patients visiting emergency departments (32%) (25). These respiratory symptoms persisted from 4 days to 4 weeks, and the duration was longer in those who had chronic symptoms at baseline (data not shown). Our finding that 37.4% of the symptomatic patients met the criteria of an acute exacerbation was consistent with the notion that viral infection could lead to acute exacerbation of bronchiectasis (8, 9, 11), possibly with secondary bacterial infection playing a role at a later stage (9, 28).

We also looked at the potential risk factors for SARS-CoV-2 infection in patients with bronchiectasis. Our survey showed that younger age and not using a bronchodilator were independently associated with SARS-Co-2 infection. There was evidence showing that patients with SARS-Co-2 infection were mostly younger than 60 years (29). Bronchodilators were recommended for patients with shortness of breath according to guidelines of bronchiectasis (15, 30–33). It was speculated that the elderly patients and patients using bronchodilators may take stricter measures for COVID-19 prevention, thus reducing the risk of being infected. For example, mask-wearing, even with the use of non-medical masks, has a substantial impact on outbreak control of COVID-19 (34). Interestingly, the odds of an individual being observed to wear a mask was higher in older adults than younger individuals (23). There are conflicting evidences on whether patients with bronchiectasis are more susceptible to COVID-19. A single-center case–control study using nationally representative data from the COVID-19 cohort and matched cohort in South Korea (20) showed that the incidence of COVID-19 was relatively higher in patients with bronchiectasis than those without bronchiectasis, and COVID-19 patients with bronchiectasis, as compared to those without, were also more likely to have pulmonary comorbidities including asthma and COPD, as well as extra-pulmonary comorbidities, such as hypertension, diabetes mellitus and heart failure. Recently, a multi- center retrospective cohort study (35) showed that bronchiectasis was not significantly associated with COVID-19 [pooled HR 0.78 (95% CI, 0.41–1.49)], but there were still no data related to the severity of the disease.

It is worth noting that most of our patients did not make medical visits but successfully managed by themselves after infection with SARS-CoV-2. Of the patients who visited hospitals, nearly 25% needed hospitalization. It was similar to a previous population cohort study in England (8,256,161 patients) showing that 25.5% of patients with chronic respiratory diseases needed to be hospitalized with SARS-CoV-2 infection, far higher than the hospitalization rate of patients with COVID-19 in the overall population (2.2%) (13). However, our study further demonstrated the necessity of health education to enhance patients’ disease awareness and self-management skills, particularly during a pandemic like COVID-19 when medical resource was allocated to emergency response.

In an outbreak of pandemic like COVID-19 when medical resources are limited, it is imperative to identify patients with exacerbated respiratory diseases who may need emergency care. Therefore, we analyzed the risk factors for emergency visit and/or hospitalization in our patients. Our results showed that these patients were more likely to be older, to have chronic symptoms of sputum production and dyspnea, to receive treatment with bronchodilators and/or expectorants, to have comorbidities including COPD and diabetes, and to have a history of acute exacerbation of bronchiectasis in the past year. A history of acute exacerbation of bronchiectasis in the past year and the use of daily expectorants were independently associated with emergency visit and/or hospitalization for patients with bronchiectasis infected with SARS-CoV-2. This result was consistent with a previous study on the impact of the COVID-19 pandemic on exacerbations and symptoms of bronchiectasis (21). The daily use of expectorants may be an indicator of frequent cough and sputum production as a manifestation of a more severe disease.

There were several limitations to our study. First, as a single-center telephone survey, the sample size was relatively small, and there may be recall bias. Second, there may be survivor bias. However, of the 995 patients who received our telephone call, 13 had died before the surge of COVID-19 in early December 2022. It is speculated that there was little impact of deceased patients on the outcomes of this survey. Third, because the patients were recruited retrospectively, and due to the time limit of a telephone survey, data related to assessment of bronchiectasis severity and etiology were not complete, such as data on the scale of dyspnea, sputum culture results, lung functions, and investigations into rarer causes for bronchiectasis which may explain the higher proportion of cases with unknown etiology in our patients. Another limitation was that of the patients who were identified as having COVID-19, 26.3% had no confirmation by a positive viral test, but only reported consistent symptoms and a history of close contact with family members with SARS-CoV-2 infection.

## 5 Conclusion

In conclusion, during the COVID-19 surge in December 2022 in Beijing, the infection rate of SARS-CoV-2 in patients with bronchiectasis was high. After SARS-CoV-2 infection, the majority of our patients experienced new-onset or exacerbation of respiratory symptoms (cough, expectoration and dyspnea) which lasted for a longer time. However, most of the patients infected with SARS-CoV-2 successfully managed at home. A history of exacerbation of bronchiectasis in the past year and daily use of expectorants were independently associated with emergency visit and/or hospitalization for patients with bronchiectasis after SARS-CoV-2 infection. Our survey results further underscore the importance of patients’ disease awareness and self-management skills during a pandemic like COVID-19.

## Data availability statement

The raw data supporting the conclusions of this article will be made available by the authors, without undue reservation.

## Ethics statement

The studies involving humans were approved by the Ethics Committee of the Peking university Third Hospital (registry M2021-428). The studies were conducted in accordance with the local legislation and institutional requirements. The participants provided their written informed consent to participate in this study.

## Author contributions

JunW: Formal analysis, Investigation, Methodology, Project administration, Writing – original draft. JR: Writing – review & editing, Data curation, Investigation. XL: Writing – review & editing, Data curation, Investigation. JuaW: Investigation, Writing – review & editing. CC: Writing – review & editing. LS: Project administration, Supervision, Validation, Visualization, Writing – original draft, Writing – review & editing. YS: Methodology, Project administration, Supervision, Validation, Visualization, Writing – review & editing.
